# Fenestration without rib resection for postoperative bronchopleural fistula

**DOI:** 10.1186/s40792-019-0629-2

**Published:** 2019-05-02

**Authors:** Masatoshi Kanayama, Yoshinobu Ichiki, Katsuma Yoshimatsu, Yusuke Takeda, Kasumi Kusanagi, Teruaki Ishida, Masataka Mori, Hiroki Matsumiya, Yusuke Nabe, Akihiro Taira, Shinji Shinohara, Taiji Kuwata, Masaru Takenaka, Ayako Hirai, Naoko Imanishi, Kazue Yoneda, Fumihiro Tanaka

**Affiliations:** Second Department of Surgery, University of Occupational and Environmental Health, 1-1 Iseigaoka, Yahatanishi-ku, Kitakyushu, 807-8555 Japan

**Keywords:** Fenestration, Without rib resection, Postoperative bronchial fistula

## Abstract

**Background:**

Fenestration is performed in patients with bronchopleural fistula to avoid a life-threatening situation. However, usually, this procedure is required 9-cm mean length of the incision with rib resection.

**Case presentation:**

A 73-year-old man underwent right lower lobectomy with lymph node dissection (ND2a-2) for primary lung cancer (cT1cN2M0 Stage IIIA) with combined pulmonary fibrosis and emphysema. He developed a bronchopleural fistula on postoperative day 20, and we performed emergency fenestration without rib resection using a Lap-protector. The patient reported minimal pain postoperatively. As the rapid deterioration of the general condition due to the recurrence of the tumor was observed at the time of his 1-year postoperative follow-up, closing of the thoracic cavity was abandoned. However, using this fenestration, the control of infection in the thoracic cavity could be sufficiently performed without complications such as pain and pneumonia, and his routine activities were unaffected postoperatively.

**Conclusion:**

Compared with conventional method, fenestration without rib resection using a Lap-protector is a more convenient and painless technique for postoperative bronchopleural fistula.

## Background

A bronchopleural fistula (BPF) is defined as an abnormal communication between a lobar or the main bronchus and the pleural space. BPF is a severe surgical complication associated with high morbidity and mortality rates [1]. Fenestration performed in patients with BPF can avoid a life-threatening situation. However, usually, this procedure is required 9-cm (range 5–16 cm) mean length of the incision with rib resection [2]. In this report, we present a minimally invasive fenestration without rib resection using a Lap-protector for postoperative bronchopleural fistula.

## Case presentation

**Fig. 1:**
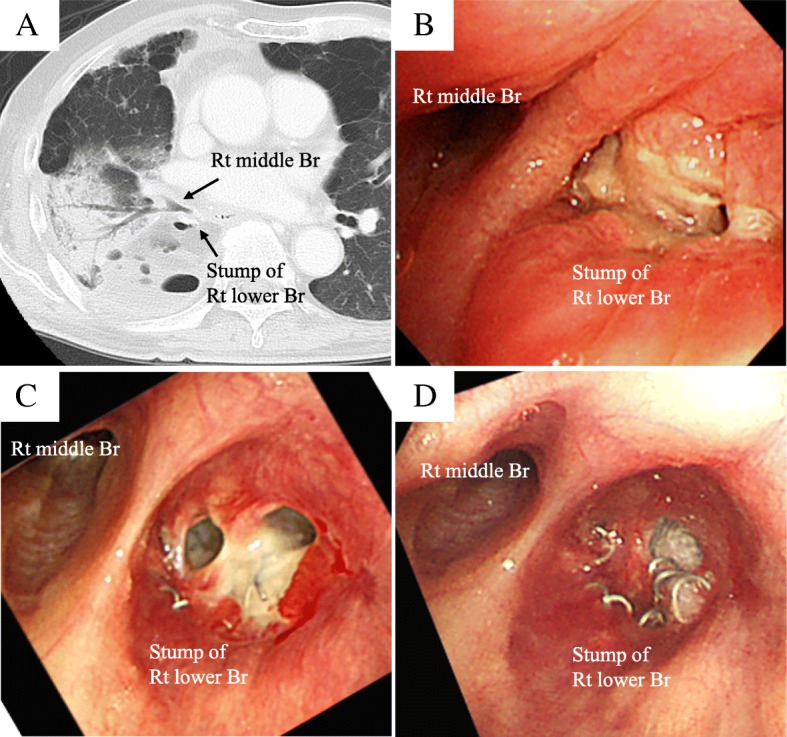
The CT revealed air retention around the bronchial stump of right lower bronchus (**a**). The image shows a bronchoscope with holes at both edges of the lower lobe bronchial stump (**b**). The image shows the bronchial stump after fenestration surgery has been performed (**c**). The image shows complete closure of the fistula did not occur by the time of his 1-year postoperative follow-up (**d**)

**Fig. 2:**
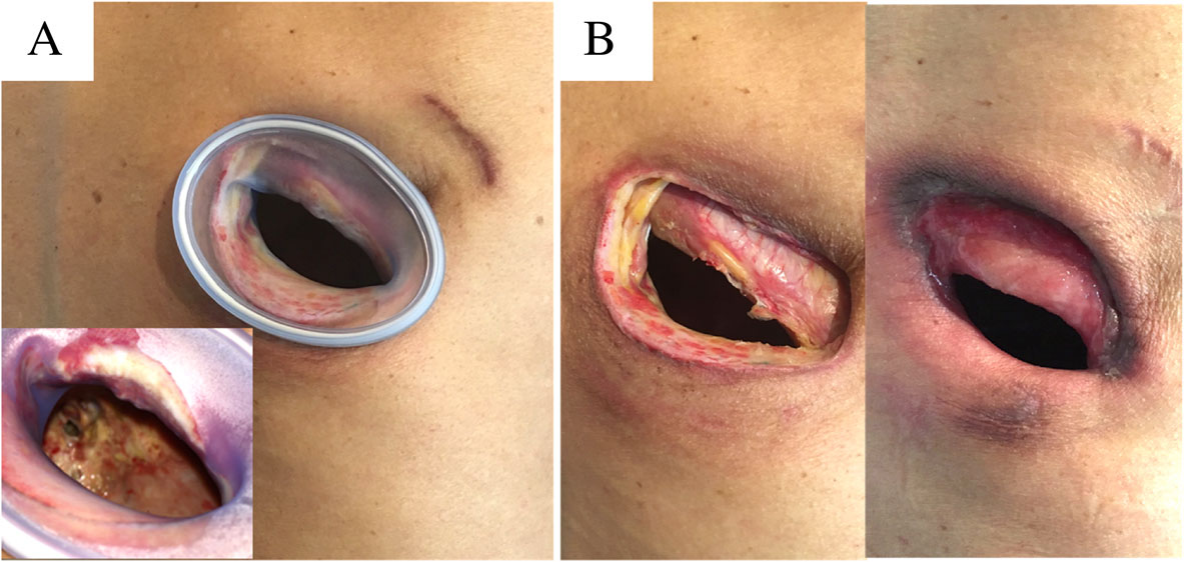
The image shows the fenestration procedure with the insertion of the Lap-protector, and the presence of the fistula is confirmed through the fenestration wound (**a**). The image shows the changes in the skin and subcutaneous tissue after fenestration surgery (**b**). Image obtained 1-month post-procedure (Left). Image obtained 6 months post-procedure (Right)

**Fig. 3:**
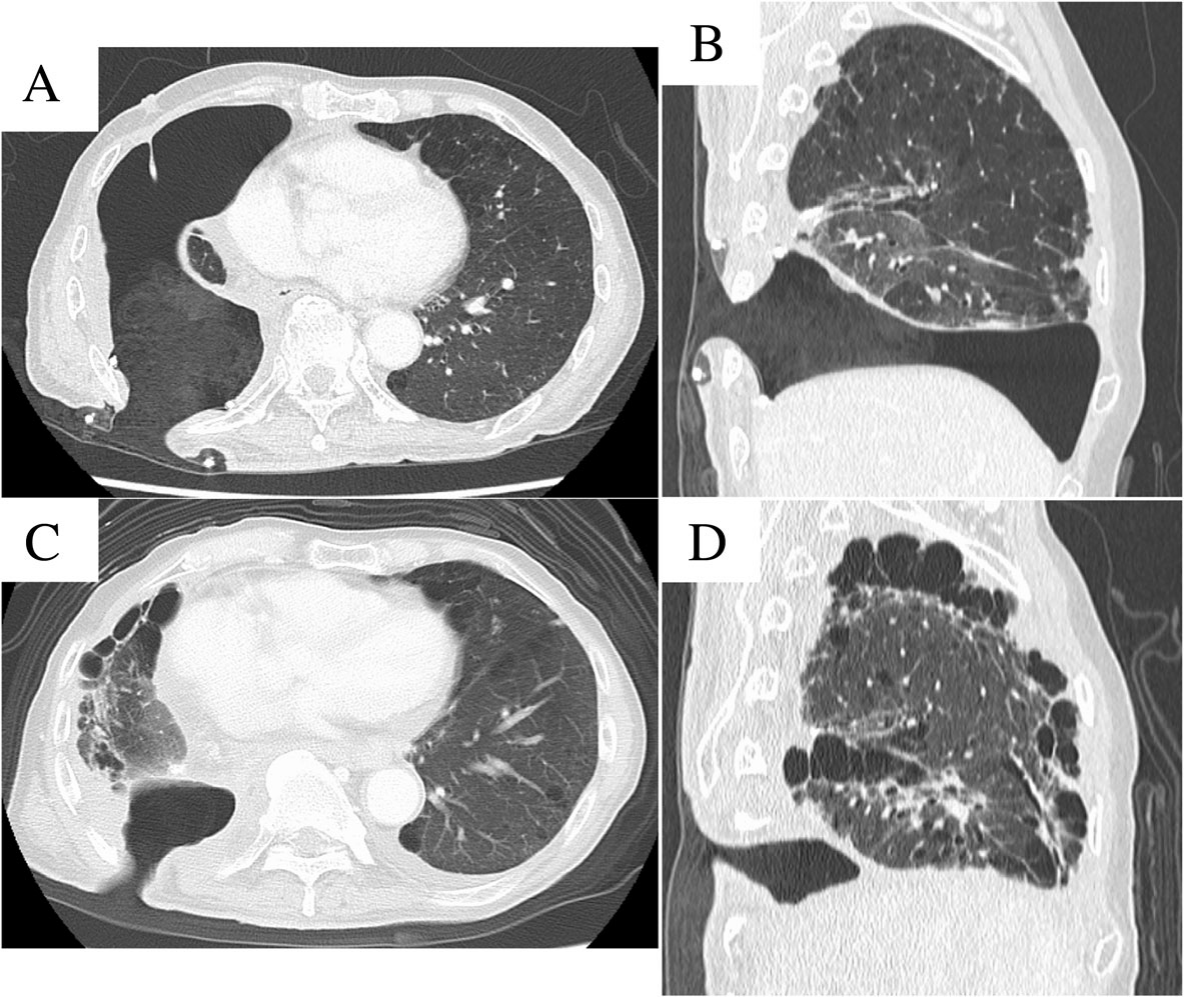
The CT shows the change of the thoracic cavity after fenestration surgery. The image obtained 1-month post-procedure (**a** axial view, **b** sagittal view). Image obtained 1-year post-procedure (**c** axial view, **d** sagittal view)

A 73-year-old man underwent right lower lobectomy with lymph node dissection (ND2a-2) for right primary lung cancer (cT1cN2M0 Stage IIIA). Because the patient was complicated with idiopathic pulmonary fibrosis with emphysema (CPFE), we performed surgery without preoperative chemoradiotherapy in consideration of the risk of acute exacerbation of interstitial pneumonia. The lobar bronchus was closed by stapler, and suture closure was performed with a pericardial fat pad graft covering the bronchial stump to prevent the development of a BPF. Overall, his postoperative course was unremarkable; however, on postoperative day (POD) 13, computed tomography (CT) revealed pneumonia of the right middle lung lobe, and antibiotic therapy was initiated. However, he was refractory to the antibiotic therapy, and repeat CT (on POD 20) revealed air retention around the bronchial stump (Fig. 1a). Bronchoscopy showed the formation of a fistula involving the bronchial stump at the right lower lobe (Fig. 1b), and he was diagnosed with a BPF. We performed emergency fenestration to control bacterial infection and avoid a life-threatening situation. Since rehabilitation was not progressed due to postoperative pain and CPFE, we decided to perform fenestration using a Lap protector to avoid extensive surgery and continue postoperative rehabilitation. Intraoperatively, we made a skin incision (6 cm in length) in the eighth intercostal space in the posterior axillary line just above the thoracic cavity and incised the subcutaneous tissue and the muscles of the chest wall. We separated the intercostal muscles and inserted a Lap-protector (FF0707, Hakko Co., Ltd, Japan) (Fig. 2a). The thoracic cavity was thoroughly irrigated, and the fistula was confirmed cranial to the pericardial fat pad graft covering the bronchial stump. Postoperatively, the daily application of gauze dressings was continued without any complications such as pneumonia, and the fenestration wound showed good healing compared with the immediate postoperative wound (Fig. 2b), his routine activities were unaffected postoperatively. Although complete closure of the fistula did not occur by the time of his 1-year postoperative follow-up (Fig. 1c, d), the bacterial infection was well-controlled and chest CT showed a prominent reduction of the thoracic cavity (Fig. 3a–d), we planned to cover the bronchial stump and to fill the thoracic cavity by omentum. However, as the rapid deterioration of the general condition due to the recurrence of the tumor was observed, closing of the thoracic cavity was abandoned, and now, symptomatic treatment is performed under the daily application of gauze dressings.

## Discussion

A Lap-protector is a device used to keep an incision open and protect the wound margins during endoscopic surgery [3, 4]. We performed fenestration without rib resection using a Lap-protector for postoperative BPF. The procedure is very simple: (1) Make a skin incision along the intercostal space. (2) Dissect the subcutaneous tissue and muscular layer (the intercostal muscles are separated in the center between the ribs to avoid exposure of the ribs). (3) Insert a Lap-protector to widen the intercostal space [5]. An advantage of this method is that it does not require resection of the ribs, unlike conventional fenestration that is performed with rib resection. Thus, this technique provides better postoperative pain control. Moreover, the fenestration wound is not exposed during insertion of the Lap-protector, and the pain associated with wound dressing changes is minimal.

Limitations of this procedure are as follows: (1) it is unclear whether fenestration without rib resection adequately controls infection associated with postoperative BPF and, (2) prolonged use of a Lap-protector may injure the skin and tissues, and leading to infection. With respect to the limitation described under no. 1, fenestration performed even without rib resection provides adequate expansion and exposure of the wound and the intercostal space. In this case, we could clearly observe the fistula and the fenestration cavity through the fenestration wound, which helped with effective drainage and infection control. With respect to the limitation described under no. 2, manipulation of a Lap-protector is associated with minimal tissue injury because the force of application is distributed equally around the incision margin [5]. Nonetheless, using too large protector leads to ischemia around the wound, and we should be concerned about the ischemic damage in the select of the optimal size protector. Moreover, the Lap-protector attachment part may easily get infected; therefore, frequent confirmation of the wound is necessary. We changed the Lap-protector on the 2nd postoperative day and confirmed no adverse event had occurred. The replacement interval was gradually extended and finally replaced at 2-week intervals. In this case, the condition of the patient’s skin and tissues was unremarkable even at the time of his 1-year postoperative follow-up. Therefore, above two limitations are acceptable and this procedure is considered to be reasonable for postoperative BPF.

Unfortunately, in this patient, closure of the BPF and the thoracic cavity were not achieved. However, using this fenestration, the control of infection in the thoracic cavity could be sufficiently performed without complications such as pain and pneumonia, and his routine activities were unaffected postoperatively.

## Conclusions

We conclude that compared with conventional fenestration with rib resection, fenestration without rib resection using a Lap-protector is a more convenient, effective, and painless technique for postoperative BPF.

## Abbreviations

BPF: Bronchopleural fistula
CPFE: Combined pulmonary fibrosis and emphysema
